# The Association of Black Cardiologists (ABC) Cardiovascular Implementation Study (CVIS): A Research Registry Integrating Social Determinants to Support Care for Underserved Patients

**DOI:** 10.3390/ijerph16091631

**Published:** 2019-05-10

**Authors:** Elizabeth O. Ofili, Laura E. Schanberg, Barbara Hutchinson, Felix Sogade, Icilma Fergus, Phillip Duncan, Joe Hargrove, Andre Artis, Osita Onyekwere, Wayne Batchelor, Marcus Williams, Adefisayo Oduwole, Anekwe Onwuanyi, Folake Ojutalayo, Jo Ann Cross, Todd B. Seto, Henry Okafor, Priscilla Pemu, Lilly Immergluck, Marilyn Foreman, Ernest Alema Mensah, Alexander Quarshie, Mohamed Mubasher, Almelida Baker, Alnida Ngare, Andrew Dent, Mohamad Malouhi, Paul Tchounwou, Jae Lee, Traci Hayes, Muna Abdelrahim, Daniel Sarpong, Emma Fernandez-Repollet, Stephen O. Sodeke, Adrian Hernandez, Kevin Thomas, Anne Dennos, David Smith, David Gbadebo, Janet AJULUCHUKWU, B. Waine Kong, Cassandra McCollough, Sarah R. Weiler, Marc D. Natter, Kenneth D. Mandl, Shawn Murphy

**Affiliations:** 1 Department of Clinical Research Center, Morehouse School of Medicine, 720 Westview Drive, SW, Atlanta, GA 30310, USA; aoduwole@msm.edu (A.O.); aonwuanyi@msm.edu (A.O.); folake_ojutalayo@hotmail.com (F.O.); jcross@msm.edu (J.A.C.); pipemu@msm.edu (P.P.); limmergluck@msm.edu (L.I.); mforeman@msm.edu (M.F.); eamensah@msm.edu (E.A.M.); aquarshie@msm.edu (A.Q.); mmubasher@msm.edu (M.M.); abaker@msm.edu (A.B.); 2 Department of Pediatrics, Duke Clinical Research Institute, Duke University School of Medicine, 2400 Pratt St., Durham, NC 27705, USA; laura.schanberg@duke.edu (L.E.S.); jhargr8175@aol.com (J.H.); adrian.hernandez@duke.edu (A.H.); thoma095@gmail.com (K.T.); anne.dennos@duke.edu (A.D.); 3 Association of Black Cardiologists,2400 N Street, Suite 200, Washington, DC 20037, USA; bhutch@ccardiac.com (B.H.); fsogade@gacri.com (F.S.); icilma.fergus@mountsinai.org (I.F.); pbduncan54@gmail.com (P.D.); jhargr8175@aol.com (J.H.); aartis@methodisthospital.org (A.A.); cvclinic4oao@aol.com (O.O.); wabat@southern-med.com (W.B.); marcus.williams.MD1@gmail.com (M.W.); david.n.smith@yale.edu (D.S.); tdgbadebo@gmail.com (D.G.); ajulujay@gmail.com (J.A.); bwaine@zoepeds.com (B.W.K.); cmccullough@abcardio.org (C.M.); 4 Department of Academic Affairs and Research, The Queen’s Medical Center, 1301 Punchbowl Street, Honolulu, HI 96813, USA; tbseto@queens.org; 5 Department of Medicine, Meharry Medical College,1818 Albion St, Nashville, TN 37208, USA; henry.e.okafor@Vanderbilt.edu; 6 RCMI Data Coordinating Center, Jackson State University, 1400 John R. Lynch Street, Jackson, MS 39217, USA; alnida.ngare@rtrn.net (A.N.); drewdentjr@gmail.com (A.D.); mohamad.malouhi@rtrn.net (M.M.); paul.b.tchounwou@jsums.edu (P.T.); jae.e.lee@jsums.edu (J.L.); traci.hayes@rtrn.net (T.H.); muna.abdelrahim@rtrn.net (M.A.); 7 Department of Biostatistics, College of Pharmacy, Xavier University of Louisiana, 1 Drexel Drive, New Orleans, LA 70125, USA; dsarpong@xula.edu; 8 Department of Pharmacology and Toxicology, University of Puerto Rico Medical Sciences Campus, P.O. Box 365067, San Juan, PR 00936, Puerto Rico; e.fernandez@upr.edu; 9 Department of Bioethics, Tuskegee University, 1200 W. Montgomery Rd., Tuskegee, AL 36088, USA; ssodeke@tuskegee.edu; 10 Department of Medicine, College of Medicine of the University of Lagos, Private Mail Bag 12003, Idi Araba, Lagos, Nigeria; 11 Department of Pediatrics and Computational Health Informatics, Harvard Medical School, 300 Longwood Avenue, Boston, MA 02115, USA; sarah_weiler@hms.harvard.edu (S.R.W.); marc.natter@childrens.hrvard.edu (M.D.N.); Kenneth.mandl@childrens.harvard.edu (K.D.M.); snmurphy@partners.org (S.M.)

**Keywords:** cardiovascular disease, registry, informatics, mobile health technology, quality care, clinical trials, post marketing surveillance

## Abstract

African Americans, other minorities and underserved populations are consistently under- represented in clinical trials. Such underrepresentation results in a gap in the evidence base, and health disparities. The ABC Cardiovascular Implementation Study (CVIS) is a comprehensive prospective cohort registry that integrates social determinants of health. ABC CVIS uses real world clinical practice data to address critical gaps in care by facilitating robust participation of African Americans and other minorities in clinical trials. ABC CVIS will include diverse patients from collaborating ABC member private practices, as well as patients from academic health centers and Federally Qualified Health Centers (FQHCs). This paper describes the rationale and design of the ABC CVIS Registry. The registry will: (1) prospectively collect socio-demographic, clinical and biospecimen data from enrolled adults, adolescents and children with prioritized cardiovascular diseases; (2) Evaluate the safety and clinical outcomes of new therapeutic agents, including post marketing surveillance and pharmacovigilance; (3) Support National Institutes of Health (NIH) and industry sponsored research; (4) Support Quality Measures standards from the Center for Medicare and Medicaid Services (CMS) and Commercial Health Plans. The registry will utilize novel data and technology tools to facilitate mobile health technology application programming interface (API) to health system or practice electronic health records (EHR). Long term, CVIS will become the most comprehensive patient registry for underserved diverse patients with cardiovascular disease (CVD) and co morbid conditions, providing real world data to address health disparities. At least 10,000 patients will be enrolled from 50 sites across the United States.

## 1. Introduction

### 1.1. Importance of the Problem and Critical Barrier to Progress on Health Disparities

The Institute of Medicine (IOM) forum on drug discovery and workshop on Public Engagement and Clinical Trials: New Models and Disruptive Technologies [1,2], identified two major barriers that support the rationale for the CVIS registry: (1) Lack of involvement of community physicians and their needs in the development and conduct of clinical trials. (2) Increasing difficulty of recruiting and retaining an appropriate human subject population for specific clinical trials. These barriers disproportionately impact minorities and individuals with limited science and health literacy, in part due to a lack of cultural competence and language sensitivity among researchers. 

### 1.2. Realizing the Full Potential of Precision Medicine in Health and Healthcare-Can We Avoid Widening Disparities? 

Precision Medicine promises to advance scientific discovery [3,4] The Precision Medicine Initiative^®^ (PMI) Cohort Program, the All of Us Research Program, aims to establish a national research cohort of 1 million or more Americans, who will share information on their health status and habits, undergo clinical evaluations, provide biospecimens, and allow access to their medical records for research. All of Us will generate an unprecedented research resource for major scientific and clinical advances, and will require a transparent process that sustains the public trust, is inclusive, flexible and resourced for sustainable participant engagement, with attention to data privacy, and novel ways of returning information to participants [5,6,7]. Barriers to clinical research may limit participation of minority patients and their physicians, as confirmed by our own focus group research [8], potentially widening disparities. All of Us current protocols do not prioritize meaningful and sustained engagement of first line practitioners, including Blacks or African Americans. CVIS will intentionally focus education and health literacy efforts on knowledge gaps in clinical research among patients and providers, including rationale and process to return research results to participants. The strategic plan for the National Center for Advancing Translational Science (NCATS) emphasized that engaging patients and their communities throughout the lifecycle of a translational research project ensures that research outcomes are relevant to and directly address patient needs and will be more readily adopted by the community [9]. Thus, CVIS will complement the All of Us Precision Medicine Initiative by addressing critical gaps in research translation that is relevant to NIH and NCATS strategic priorities.

### 1.3. Increasing Costs and Complexity of Drug Discovery and Development

U.S. clinical trials are more expensive [10], and inefficient with 90 percent of launched trials failing to meet enrollment goals; 5–20% do not enroll a single patient. On average, it takes over 10 years for a new medicine to go from initial discovery to patients at an average cost of $2.6 billion in 2013, with another $312 million for post-approval research [10]. The average per patient costs of industry sponsored trials is $36,000, and participant recruitment consume up to 40% of the budget. Recruitment costs increased 58% from 2003 to 2011 [11]. Even the NIH offshores clinical trials [12,13], despite concerns about generalizability of results to the US population.

Globalization of the clinical trial enterprise (CTE) potentially impacts the quality of healthcare and widens health disparities. Clinical trials should include participants from across the demographic spectrum [14]. As noted by FDA Commissioner Margaret Hamburg (August 2014), “a guiding principle of the FDA’s goal to meet the health needs of patients across the demographic spectrum, is the importance of diversity in clinical trials” [15]. The FDA Safety and Innovation Act (FDASIA) of 2012 [16] includes a requirement to study the availability of data on the participation of demographic subgroups (sex, age, race, and ethnicity) in clinical trials [17] A recently approved drug for heart failure illustrates the challenge—only 5% of the 8400 study participants were enrolled at sites in the United States (U.S.). Blacks in the trial suffered more angioedema in the treatment arm (2.4%) with a tenfold increase among U.S. Blacks (5.6% vs. 0.5%) for the overall study. This finding limits generalizability of this recently approved drug to black patients, thus potentially impacting care, and exacerbating existing disparities in heart failure [18].

Barriers to clinical research participation are at multiple levels: the individual (participant/ patient), provider (physician/health system) and community [19,20,21,22,23,24,25,26,27,28,29,30,31,32,33,34]. There are multiple reasons why minority patients do not participate in clinical research: mistrust of the provider/health system/research team [19]; lack of information [20]; fear of harm/side effects [21]; complex protocol; health related factors/co-morbidities [22]; not setting targets [23]; not being asked [24]; provider or researcher attitude [24]. Provider barriers include lack of infrastructure in the practice, lack of training/knowledge [25]; not familiar with investigators [26]; afraid of losing patients; time away from practice; poor communication by the research team. Community barriers include lack of information/readiness of community leaders [27,28,29,30], and cultural incompetence or lack of sensitivity of researchers to the needs of minority communities [31,32,33,34]. 

### 1.4. Knowledge Gap

There are no data, to indicate if physicians’ concerns about eligibility of minority patients are due to their experience with patients’ comorbidities, beliefs around health literacy, protocol compliance, or some combination of these factors. The IOM Roundtable, titled Promotion of Health Equity and the Elimination of Health Disparities-Strategies for Ensuring Diversity, Inclusion, and Meaningful Participation in Clinical Trials (IOM 2016) [15] identified the following gaps: (1) Lack of transparency and information sharing between researchers and patients and referring community physicians (2) A need for evidence generation and metrics for participant engagement and trust in the research process, including informed consent. 

## 2. Materials and Methods

### 2.1. Research Hypothesis 

Using a combination of Medidata Cloud-based technology for electronic data capture and i2b2/SHRINE federated clinical research data platform, the CVIS Registry will collect at least 10 years of comprehensive clinical practice data, including detailed safety and treatment data, on underserved patients who have co morbid cardiovascular disorders, leading to understanding how to best apply available therapies while improving the real world practice setting for clinical trials. Further, we hypothesize that a mobile health application interface (API) will enable patient engagement in clinical care and returning research results, while facilitating new efficiencies in the administration of surveys and patient-reported outcomes (ePROs) for adaptive clinical trials. 

### 2.2. Approach- Collaborating Partners

#### 2.2.1. The Association of Black Cardiologists 

Founded in 1974, the Association of Black Cardiologists, Inc., (ABC) (www.abcardio.org) is a nonprofit organization with an international membership of over 1800 health professionals, lay members of the community (Community Health Advocates), corporate members, and institutional members. The mission of the ABC is to promote the prevention and treatment of cardiovascular disease, including stroke, in Blacks and other minorities, and to achieve health equity for all. The ABC is dedicated to eliminating the disparities related to cardiovascular disease in all people of color. Today, the ABC’s public and private partnerships continue to increase its impact in communities across the nation. The Association of Black Cardiologists, Inc. is fully accredited by the Accreditation Council for Continuing Medical Education (ACCME). Acting on its longstanding desire to establish a member enabled clinical registry that will simultaneously address gaps in clinical practice and research, ABC President Dr. Icilma Fegus recruited Elizabeth Ofili, MD, MPH to develop the ABC CVIS registry in collaboration with academic health centers and research organizations [35]. The registry was supported by subsequent ABC leaders Drs. Barbara Hutchinson and Felix Sogade, and approved by a unanimous vote of the Board of Directors, as a long-term strategic initiative that will establish the most comprehensive patient registry for minority patients with CVD and co morbid conditions, and provide real world data to address health disparities. The registry will serve as a foundation for future studies including quality improvement, adaptive clinical trials, implementation research, and pharmacosurveillance. ABC member practices are eligible to join the CVIS registry, and enroll diverse patients from their respective practices.

#### 2.2.2. Morehouse School of Medicine (MSM) 

Located in Atlanta, Georgia, Morehouse School of Medicine (MSM) (www.msm.edu) is among the nation’s leading educators of primary care physicians and was recognized in 2010, as the top institution among U.S. medical schools for achieving a social mission. The National Center for Primary Care (NCPC) at MSM has the unique distinction of being the only Congressionally-sanctioned center in the country dedicated to promoting excellence in community-oriented primary health care and optimal health outcomes for all Americans, with a special focus on underserved populations and on the elimination of health disparities. MSM sponsored clinical and translational research infrastructure will provide clinical coordinating support for the ABC CVIS Registry.

#### 2.2.3. The Georgia Clinical and Translational Science Alliance (GaCTSA) 

Created in response to the NIH CTSA program, the Georgia Clinical & Translational Science Alliance (GaCTSA) (www.gactsa.org) serves as a catalyst and incubator for clinical and translational research across Georgia and with regional and national impact. GaCTSA is a compelling partnership of Emory University, Morehouse School of Medicine, the Georgia Institute of Technology and the University of Georgia. GaCTSA healthcare partners are: Emory Healthcare, Morehouse Healthcare, Children’s Healthcare of Atlanta, the Atlanta VA Medical Center, the Grady Health System, and the Atlanta Community Physicians Network and translational science partners including the Yerkes National Primate Research Center (located at Emory), the Georgia Research Alliance, Georgia Bio and links to the Prevention Research Centers of the US Centers for Disease Control and Prevention (CDC). Built over the past decade of collaboration, the partners of GaCTSA have forged strong research networks and GaCTSA has become a “24 hour” home and catalyst for innovative, high quality clinical and translational research with local, national and global impact [36,37].

#### 2.2.4. Clinical Research Center 

The MSM campus includes one of the few free- standing, JACHO-accredited Clinical Research Centers (CRC, approximately 12,000 sq ft). The CRC has five fully equipped examination rooms; shared use laboratories; 16-bed study participant observation unit; state-of- the-art emergency alert system; cardiovascular laboratory (equipped with echocardiography, vascular ultrasound and vascular compliance instruments); negative-pressure Isolation exam room and kitchen. The CRC provides shared resources in: research subject recruitment, analytical laboratory, nutrition core (with nursing and dietician), research nurses, research assistants, consultation for IRB applications, bioinformatics, biostatistical support, as well as clinical trial design. The MSM CRC has an excellent track record in organizing a wide variety of outreach programs in minority communities that promote the involvement of minority participants in clinical studies [38]. The Community Advisory Board (CAB) includes community leaders and has further strengthened MSM-community interactions and enhanced these recruitment efforts. The Community Physicians Network (CPN), is a practice-based consortium that has grown to a diverse group of 150 primary care practices, subspecialty and surgical practices with over 350,000 annual out-patient visits throughout metro-Atlanta, Macon and Columbus. Georgia. Moreover, the creation of this unique resource provides an exciting opportunity to conduct clinical research with high-risk populations in the “real world” setting of ethnic communities. The CPN was established to address disparities in health care by focusing on provider-specific strategies, increased participation in clinical trials of effective therapies by African Americans and other underrepresented minorities and diminishing barriers in the healthcare delivery system resulting in poor access to care. CPN is an AHRQ designated Practice Based Research Network with a good track record both in Quality Improvement activities at the practice level as well as facilitating improvements in self-management skills among patients with chronic diseases [39,40]. 

#### 2.2.5. Mobile Clinical Research Vehicle

The mobile clinical research vehicle is a 30 ft. self-contained, handicap accessible, mobile research facility that can be used to conduct clinical research. The mobile unit allows MSM researchers to reach people in rural areas and those unable to come to the center, diverse populations and visitors to various Atlanta venues are able to join clinical research studies, as well as learn about various health disparities that still plague our communities. It contains two exam rooms, a laboratory, private areas for patient interviews, restroom, and audio/visual technology for patient education. Onboard equipment includes cardiac monitors, exercise treadmill, cardiovascular ultrasound, refrigerator and freezer for specimen processing, scale and computers with internet access.

### 2.3. RCMI Translational Research Network 

The Research Centers at Minority Institutions (RCMI) Translational Research Network (RTRN) (www.rtrn.net) and its Data Coordinating Center (DCC) provides support to investigators across RCMI institutions. RCMI and RTRN have established a track record in addressing health disparities and workforce diversity through strategic partnerships that support research collaboration [37,41,42,43,44]. 

The current staff of the DCC Biostatistics Division includes three senior members. They have 50 years of combined statistical experience and led/coauthored over 160 peer-reviewed scientific articles. They have supported various research projects including animal experimental study, genetics, clinical trials and epidemiology/community study. They provide expertise in study design, parametric and nonparametric longitudinal data analysis, latent variable analysis, outcome measurements, medical decision analysis, and Rasch psychometric analysis, statistical methods for assessing gene-environment interaction, clinical trial design, computing power and sample size for correlated samples, multiplicity adjustment for confidence intervals, risk behavior (HIV, drug abusing, smoking, etc.), cardiovascular disease, quality of life research, evaluation of intervention programs, cardiovascular disease epidemiology, and pharmacoeconomics and outcomes research, etc. The DCC is co-located with Venture’s Data Center at the Mississippi e-Center, and provides data management services to Morehouse School of Medicine and RCMI institutions, using Venture’s industry-grade data security resources. Morehouse School of Medicine and the RTRN Data Coordinating Center will provide Clinical Coordinating and Data Coordinating support respectively, for ABC CVIS Studies.

### 2.4. Duke Clinical Research Institute (DCRI)

DCRI has a track record of designing and implementing innovative clinical trials grounded in the realities of patient care. DCRI is also houses more registries than any organization except the NIH. DCRI expands the impact of clinical research beyond regulatory approval by designing trials that advance fundamental understanding of health and disease and inform efforts to improve the quality of care. DCRI is home to the Childhood Arthritis and Rheumatology Research Alliance (CARRA) Registry for Childhood Rheumatic Disease [45,46,47,48]. The ABC CVIS design and i2b2 informatics infrastructure is modeled after the CARRA Registry and Laura Schanberg, MD, CARRA Registry principal investigator, and Adrian Hernandez, MD, Associate Director of DCRI, provide ongoing consultation and training support to the ABC CVIS Registry team.

### 2.5. Informatics Infrastructure Support for the Registry

#### 2.5.1. Informatics for Integrating Biology and the Bedside (i2b2) and SHRINE (Shared Health Research Informatics Network) 

i2b2/SHRIE are informatics innovations, funded by the National Center for Biomedical Computing (NCBC) and NIH, and based at Partners Health Care System in Boston. i2b2/SHRINE enables effective collaboration for precision medicine, through the integration, standardization, sharing and analysis of heterogenous data from healthcare and research as well as through engagement and mobilization of a life sciences focused open-source, open-data community [49,50,51,52,53]. Bioinformatics teams at MSM and RTRN Data Coordinating Center have expertise in implementing i2b2/SHRINE, and will support this effort in collaboration with the informatics team at Harvard Medical School.

#### 2.5.2. Medidata Rave Electronic Data Capture (EDC) 

Medidata Rave Electronic Data Capture (EDC) [54] captures, manages and reports registry and clinical trial data. This FDA 21CFR11 compliant, cloud-based technology will support ABC CVIS Registry from randomization, to site data entry, medical coding, safety reporting and mobile health capabilities, with flexibility for adaptive trial designs, and remote monitoring.

#### 2.5.3. Health 360x-A Mobile and Web based Patient Engagement Platform 

Health 360x is an engagement and health communications platform that has successfully integrated health coaching intervention with self-monitoring, in patients with chronic disease.

At least 400 predominantly African American patients with multiple chronic conditions, have successfully used the technology to self monitor and manage their health. [55,56,57,58,59]. ABC CVIS will use the Health 360x or similar engagement and analytic platform, to implement a seamless and secure access to practice electronic health record (EHR) using SMART on FHIR standardization.

#### 2.5.4. Substitutable Medical Applications and Reusable Technologies (SMART) 

SMART (Substitutable Medical Applications and Reusable Technologies) defines an open application programming interface (API) specification that enables mobile applications to connect to electronic health record systems using FHIR (Fast Healthcare Interoperability Resource) specification [60]

#### 2.5.5. SMART on Fast Health Interoperable Resources (FHIR) 

This is a health IT system that has implemented the SMART on a FHIR specification, including SMART’s profiled versions of FHIR; currently, FDA-funded efforts are in progress to expand FHIR for 21 CFR 11-compliant safety reporting for registries [61,62] Health 360x will standardize on SMART on FHIR specification, to enable data sharing with practice sites’ EHR, as well as allow patients and research volunteers to upload and transmit their health information, including patient reported outcomes and SF-12 quality of life and similar surveys [63,64,65]. Clinicians and members of the research team can use secure electronic messaging to communicate with patients, share informed consent and health/research information [66].

### 2.6. Vanguard Site Principal Investigators

Barbara Hutchison, MD; General and Noninvasive Cardiology Practice, Annapolis, Maryland.

Felix Sogade, MD; Cardiac Arrhythmia and Heart Failure Practice, Macon, Georgia.

Joe Hargrove, MD; General and Interventional Cardiology Practice, Little Rock, Arkansas.

Philip Duncan, MD, General and Interventional Cardiology Practice, Richmond, Virginia.

Osita Onyekwere, MD; General and Interventional Cardiology Practice, Anniston, Alabama.

Andre Artis, MD; General and Interventional Cardiology Practice, Gary, Indiana.

Adefisayo Oduwole, MD; General and Invasive Cardiology, Morehouse School of Medicine, Atlanta.

Stephanie Kong, MD and B. Waine Kong, PhD; Zoe Pediatrics, Thomaston and Columbus Georgia.

David Gbadebo, MD, Electrophysiology Practice, Decatur, Georgia.

David Smith, MD, Cardiology Practice, Yale Medical Center.

Henry Okafor, MD, General and Noninvasive Cardiology, Meharry Medical College, Nashville TN.

Todd Seto MD, General Cardiology, University of Hawaii, at Manoa, Honolulu, HI.

**Figure 1:**
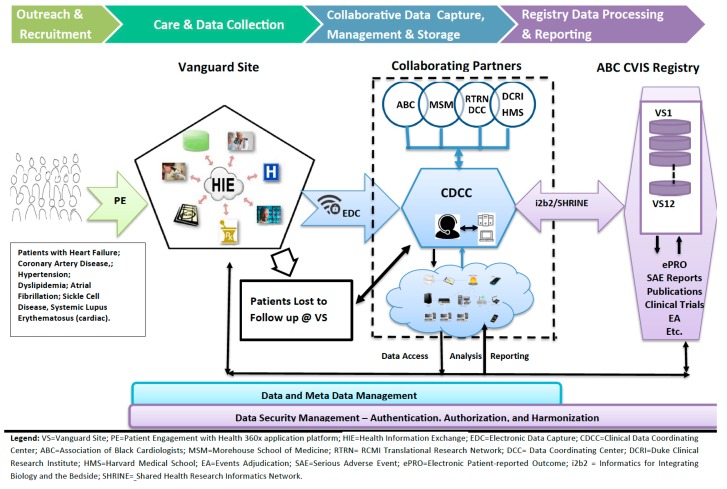
ABC CVIS Registry Schema.

Eligibility and Main Inclusion Criteria: (1). Eligible patients must be receiving clinical care at participating ABC member practices. The Registry protocol will be revised and sample size estimates adjusted, based on cardiovascular diseases that are prioritized for inclusion (see Figure 1, registry schema). (2). Subject and/or parent/legal guardian is able to provide written informed consent and willing to comply with study procedures. 

Primary Study Objective: (1) Prospectively collect socio-demographic, clinical and biospecimen data from enrolled adults, adolescents, and children with prioritized cardiovascular diseases. (2) Evaluate the safety of new therapeutic agents or devices, in enrolled participants.

Secondary Objectives: (1). Evaluate clinical outcomes associated with the use of therapeutic agents. (2). Document drug/device treatment patterns over time. (3). Evaluate factors other than drug treatment, such as social determinants, that are associated with clinical outcomes. (4). Support enrollment in NIH and industry sponsored clinical trials, including adaptive designs. (5) Support implementation science research that require real world data. (6) Support comparative effectiveness research. (7) Support CMS and Health Plans quality care programs.

Treatment Regimen: Patients will receive diagnostic, preventive and therapeutic interventions per standard of care.

Duration/End of Study: This study will continue as long as the Registry remains in existence with a goal of 10 years of follow-up for each subject. The long-term follow-up program may allow for data collection beyond 10 years of follow-up.

Number of Subjects: Target 10,000 eligible subjects enrolled at ABC CVIS Study sites with no maximum number of subjects/site. Target numbers may exceed 10,000 with IRB approval.

Number of Sites: No fewer than 50 participating clinical sites in North America (with the addition of international sites from Sub Saharan Africa in the future). The principal investigator at each participating clinical site will be an ABC member in good standing. Vanguard sites will enroll the first 2000 subjects.

### 2.7. Study (Registry) Design 

See Figure 1 for CVIS schema. This is an observational cohort study of adults and children with prioritized cardiovascular disease receiving care at participating sites. All patients with prioritized diseases, who are seen at a participating site for medical care will be considered for enrollment into the registry. Patient engagement (PE), participant recruitment and retention at each Vanguard site (VS) will be supported by Health 360x Mobile and web-based platform. Health 360x disease specific coaching will also enable patient reported outcomes (ePRO) [55,56,57,58,59] for quality improvement and adaptive clinical trial design. Patients are seen at baseline and then at least twice a year, and also when new disease specific drugs or devices are prescribed or implanted. Data will be collected longitudinally from enrolled subjects (via paper or online surveys, personal interview, and/or phone or electronic contact). Data may be collected from the participant’s medical record or electronic health record (EHR) and Health Information Exchange (HIE) at any time during participation in the ABC CVIS. Data collection may also be supplemented by linkage to administrative claims data or from other sources such as regional cancer registries, AHA Get with the guideline registries, device registries, the National Death Index, claims databases maintained by government and commercial insurance providers, and other external data sources (e.g., health plans and health information networks, pharmacy records, laboratory test results, other patient registries). These linkages will be performed using identifying information, such as name, DOB, address, and SSN (if provided). These data will be sufficient for the identification of safety signals and for the conduct of rigorous pharmacoepidemiologic safety studies. This protocol will be conducted in accordance with the policies and procedures of the FDA guidance document: Good Pharmacovigilance Practices and Pharmacoepidemiologic Assessment, March 2005, and capture data in a secure manner, compliant with the Health Insurance Portability and Accountability Act (HIPAA). 

Essential data elements—including demographics, social determinants, disease phenotype: heart failure severity e.g., ejection fraction, disease activity (SLE; sickle cell), and cumulative medication exposures—will be obtained from all participants at enrollment and approximately every 6 months as standard of care dictates during routine follow-up. Data will also be collected whenever treatment with new disease specific drugs or devices are prescribed or implanted. Serious adverse events (SAEs) as defined by the FDA and protocol-defined medical events of special interest (ESIs) will be prospectively reported to the CDCC by investigators at the time of the event. ESIs will be verified against medical records, such as hospital discharge summaries. Selected safety events will be adjudicated by a panel of physicians who are content experts via review of medical records from which identifying patient information has been redacted. 

Data analysis practices and procedures will be guided by the International Society for Pharmacoepidemiology Guidelines for Good Pharmacoepidemiology Practice [67]. Detailed analysis plans will be developed before each data analysis. Appropriate study designs will be used to estimate associations between medication exposures and SAEs and ESIs. It is anticipated that cohort study designs will be used for most analyses, but case-control, nested case-control, case-crossover, or other study designs may be appropriate to answer some questions. Comparators will be derived from subjects enrolled in the ABC CVIS Registry who were not exposed to the medication(s) of interest. Comparators may be further defined by specific medication exposures or disease state. Operational definitions of cohorts, medication exposures, confounders, etc., will be determined based on the specific study question. Confounding and effect modification will be considered in statistical models. Relevant sensitivity analyses, particularly of medication exposure risk windows, will be performed.

#### Informed Consent and Enrollment

Patients with prioritized diseases will be offered the opportunity to be enrolled into the ABC CVIS. Each participant for whom written or electronic informed consent is obtained and who meets all eligibility criteria will be enrolled in the ABC CVIS. At the time of enrollment, participants and/or parent/legal guardian will be asked to provide permission for a continuing release of medical records to facilitate procurement of documentation of safety events that may occur. [68]. Each participant will be offered access to Health 360x mobile and web-based application, with coaching support to facilitate ongoing engagement for quality improvement and patient reported outcomes. 

#### Data Collection Process 

Data will be collected directly from participants (via paper or electronic questionnaires and/or personal interview) when participants visit an ABC CVIS study site, or remotely. When necessary, participants may be contacted, or initiate contact themselves, via phone, mail or electronically by site staff or a call center to facilitate data collection. Appropriate protections of personally identifying information will be employed for all participant contact. Data collection may be supplemented by data extracted from the participant’s medical record or EHR from dates on, before, between, and subsequent to baseline and follow up visits. Data may be collected from the participant’s medical record or electronic health record (EHR) at any time during participation in the ABC CVIS. Data collection may also be supplemented by linkage to administrative claims data or from other sources such as regional cancer registries, AHA Get with the guideline registries, device registries, the National Death Index, claims databases maintained by government and commercial insurance providers, and other external data sources (e.g., health plans and health information networks, pharmacy records, laboratory test results, other patient registries). These linkages will be performed using identifying information, such as name, DOB, address, and SSN (if provided). Data will continue to be collected for the duration of participation in ABC CVIS. 

#### Long Term Follow up

Participants may discontinue care at ABC CVIS sites for many reasons, including relocation. When this occurs, participants will remain in the ABC CVIS through a systematic long-term follow-up program to which they consent to participate in at the time of enrollment. Health 360x mobile and web-based application will support ongoing participant engagement and long term follow up. 

#### End of Study or Withdrawal 

Long-term follow-up will continue for at least 10 years; however, participants or legal guardians can withdraw participation in the ABC CVIS at any time during the study, for any reason, without consequence. No special assessments will be required if the Registry is discontinued or a patient and/or parent/legal guardian opts to discontinue participation. If withdraw consent for study participation entirely, they will be free to do so. Data collected prior to the withdrawal of consent will remain in the Study. Site staff will indicate the date and reason for withdrawal in the eCRF. 

#### Data and Meta Data Management 

The flow of data from initial participant engagement (PE) and recruitment will utilize interoperable mobile and web applications such as Health 360x, SMART on FHIR to minimize burden on practice for ongoing data collection. Electronic Data Capture (EDC) will utilize Medidata RAVE (see Figure 1). The Clinical Data Coordinating Center (CDCC) will be operationalized with the support and expertise of collaborating partners. Similarly, practice site specific federated data access will be accomplished using i2b2/SHRINE [49,50,51,52,53]. 

Each ABC CVIS site PI will be able to access his/her site’s data over an encrypted, secure connection using a unique username/password-based authentication. Authorized investigators with appropriate access verified by their unique username/password combination will also be allowed to view statistical measures and aggregate information summarized across ABC CVIS sites using i2b2/SHRINE query tool, which allows federated access. 

#### Roles of Collaborating Institutions and Organizations 

The Association of Black Cardiologists (ABC) is a primary sponsor of the ABC CVIS registry, and has responsibility for fundraising and broad outreach to its members for participation in the registry. Morehouse School of Medicine (MSM) is a co-sponsor and has responsibility for scientific direction of the registry and fundraising. MSM will house the Clinical Data Coordinating Center (CDCC), and provide support for training of clinical research coordinators as well as informatics support for i2b2/SHRINE in collaboration with the RTRN DCC. The RCMI Translational Research Network (RTRN) Data Coordinating Center (DCC) at Jackson State University will contribute infrastructure and personnel support to the CDCC at MSM, including i2b2/SHRINE query of ABC member practices. The Duke Clinical Research Institute (DCRI) and Harvard Medical School (HMS) will provide consultation, scientific and information technology expertise for the CDCC and i2b2/SHRINE implementation. Researchers at HMS pioneered electronic medical records (EMR) data integration with i2b2/SHRINE, SMART and FHIR and will contribute expertise in informatics, post-marketing research and surveillance. 

Data Collection via Electronic Case Report Forms. The EDC process consists of direct data entry at the study sites into the EDC system(s) provided by the CDCC. Sites will enter data into eCRFs according to eCRF instructions provided and project-specific training. The investigator at each clinical site is responsible for maintaining accurate, complete and up-to-date records on his/her participants, and for ensuring the completion of the eCRFs. 

#### Patient Reported Outcomes (PROs) Data Collection 

PROs will be obtained utilizing multiple modalities, including via paper (RedCAP), electronic/Mobile, and telephone surveys, with electronic data entry by subjects preferred. Participants or their parents/guardians will complete PROs either at the time of clinical research visits, periodically at other scheduled times from home, or ad-hoc related to the occurrence of events of interest. Paper-based PRO data will be entered into the EDC system by site or CDCC staff, as will data manually collected during telephone surveys by the CDCC Call Center. Electronic surveys will be stored in the respective electronic PRO system databases and then uploaded into the appropriate i2b2 data mart. Participants or their parents/guardians will be informed to communicate directly with the treating physician about any specific problems or care issues that may arise. 

#### Training Sessions and Procedures 

Training is an important method for ensuring study procedures are performed consistently, accurately and reliably. Site training is a primary component of this registry study and will include Investigator and Study Coordinator meetings held at least yearly via the web or face to face, teleconferences, study newsletters and a manual of operations, which will form the basis for site training and education. The CDCC will maintain records of all CDCC initiated training.

Training sessions will be repeated periodically as required over the course of the study. 

#### Analysis Plan 

This prospective observational registry will collect essential data in adults, adolescents and children with priority diseases who are enrolled at ABC CVIS sites, irrespective of treatment. The goal duration of follow-up is 10 years for each patient. Descriptive statistics will be used to describe ABC CVIS, and evaluate the demographic and clinical characteristics of subjects, medication usage, incidence rates of SAEs and ESIs, and regional differences. In addition, the collected data will be sufficient to perform rigorous pharmacoepidemiologic safety studies of SAEs and ESIs. The aim of these analyses will be to estimate associations between individual therapeutic agents and selected uncommon and delayed SAEs and ESIs, with consideration of confounding factors and effect modifiers. Data from ABC CVIS will be used for a variety of different projects. Each project will develop its own statistical plan based on proposed use. 

#### Statistical Design 

ABC CVIS will collect essential data elements about disease phenotype, patient characteristics, medication use, and SAEs and ESIs for each subject as outlined. Target sample size is 10,000, however, there is no maximum sample size; all eligible persons are candidates for inclusion in the safety registry. 

## 3. Discussion

This baseline methodology paper describes the rationale and design of the ABC CVIS registry. The design of the registry addresses gaps in the current evidence base for cardiovascular care. Initial priority conditions for the registry include diseases, such as heart failure and atrial fibrillation that have significant disparities in cardiovascular care. Both conditions have recently received approval of novel drug therapies, however the clinical trials did not include a sufficient number of Black patients. This lack of evidence leaves practicing physicians struggling to determine if these drugs are more effective than existing treatments in this patient population. Similarly, the presence of multiple comorbidities and social determinants may lead to drug interactions and more adverse effects. The ABC CVIS registry design will provide data that should address these evidence gaps. Perhaps, most relevant to the ability to engage, recruit and retain busy cardiology practitioners and their patients, the ABC CVIS Registry, will use information technology tools such as Health 360x, SMART on FHIR and i2b2/SHRINE to reduce the practices’ burden for ongoing participation. Additionally, integration of mobile technology is designed to increase patient engagement, by keeping them returning for appointments and “stickiness” to the practice. Patient engagement will increase overall patient satisfaction, and potentially improve patient and practice adherence to preventive care, which are quality measures for payment/reimbursement by the Centers for Medicare and Medicaid Services (CMS) and Commercial Health Plans. 

**Figure 2:**
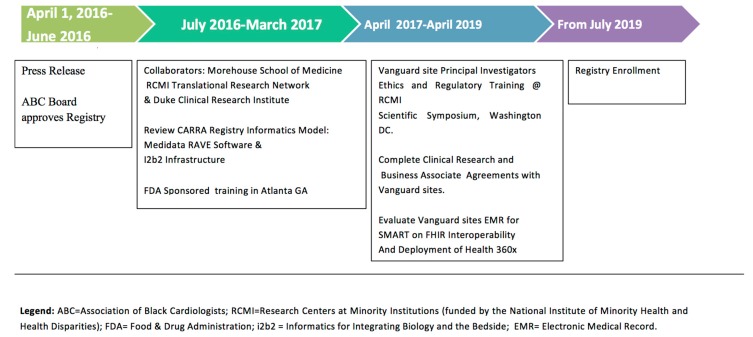
ABC CVIS Registry Timeline.

It is very significant that the registry design will focus on pharmacovigilance [67], including standardized data collection for monitoring adverse events and events of special interest. ABC CVIS was launched with training of 10 Vanguard sites in March 2017. This FDA sponsored training event attracted over 150 multidisciplinary clinicians, investigators and community partners for a full day workshop focused on Diversity and Inclusion in Clinical Trials [68]. A regulatory (Single IRB) and ethics workshop for Vanguard sites was also conducted at the RCMI Translational Science Conference in November 2017 [41]. Vanguard sites and study teams are currently completing study agreements, and assessment to confirm that their practice electronic medical records can be accessed with registry tools such as i2b2/SHRINE, SMART on FHIR mobile technologies for efficient data collection. Figure 2 shows the project timeline from press announcement [35], through anticipated start up in July 2019.

Training of 12 Vanguard Practice site principal investigators and research coordinators will be conducted at the Association of Black Cardiologists Annual Scientific Conference (Spring 2019). We anticipate enrolling the first patients by July 2019.

## 4. Conclusions

The ABC CVIS Registry is a novel study that takes advantage of current advances in health information technology, and the universal adoption of electronic medical records, to incorporate busy practitioners, in generating real world evidence that will guide appropriate treatment of patients. Another potential benefit is that these practices can enhance their capacity to participate in clinical research studies sponsored by industry and the National Institutes of Health. The collaboration between the Association of Black Cardiologists, Morehouse School of Medicine and the RCMI Translational Research Network is relevant to the focus on minority health and underserved patients and should lead to a sustained impact of the registry.

## In Memoriam

In Loving Memory of Mrs. Cassandra D. Jackson, Former Program Manager, RCMI Translational Research Network Administrative Core at Morehouse School of Medicine (30 May 1962–8 August 2018).
